# Management of an outbreak of invasive group A *Streptococcus* in a rural Australian residential aged-care facility, 2023

**DOI:** 10.5365/wpsar.2025.16.3.1176

**Published:** 2025-09-01

**Authors:** Hannah Woodall, Teresa McGorm, Rikki Graham, Amy Jennison, Priya Janagaraj

**Affiliations:** a Darling Downs Health, Toowoomba, Queensland, Australia.; b Griffith University, Gold Coast, Queensland, Australia.; c Public Health Microbiology, Public and Environmental Health, Pathology Queensland, Queensland Health, Brisbane, Queensland, Australia.

## Abstract

**Objective:**

To outline the management of an outbreak of invasive group A Streptococcus (iGAS) in a residential aged-care facility in rural Queensland, Australia, comparing outbreak management with the newly released Australian Series of National Guidelines (SoNG) for this disease and exploring unique aspects of rural iGAS outbreak management.

**Methods:**

An outbreak of iGAS was identified in a rural Queensland residential facility, where two cases occurred within 24 hours. A confirmed case was defined as any individual linked to the facility who had laboratory evidence of group A Streptococcus (GAS) in a sterile site. Whole genome sequencing was performed on all confirmed cases. The public health management of this outbreak was conducted according to the Queensland Communicable Disease Control guidelines and was compared with the new SoNG.

**Results:**

A phylogenetic tree confirmed that the two samples clustered closely together with a single allele difference. Chemoprophylaxis was offered to all residents and staff in the affected part of the facility; 95% (42/44) of residents consented to chemoprophylaxis. Increased surveillance for GAS and increased facility cleaning were recommended by the public health unit. No additional cases were identified after 30 days of surveillance. Management of the outbreak largely aligned with the SoNG except for post-outbreak surveillance, which would have been extended under the new guidelines.

**Discussion**

This paper highlights factors unique to managing iGAS outbreaks in rural areas. Rural workforce factors and access to pathology services impact rural outbreak management, and thus involving local services and considering the local context are vital. The use of chemoprophylaxis continues to be recommended by the SoNG, and in this case was considered to be an important adjunct to other management strategies.

Group A *Streptococcus* (GAS) is a Gram-positive bacterium that causes a wide variety of clinical manifestations, ranging from asymptomatic infections to invasive disease that causes bacteraemia, sepsis or toxic shock syndrome. (*1*) GAS is spread by person-to-person transmission, most commonly via droplets or broken skin. (*2*) Each year, GAS causes more than 18 million serious complications and up to 500 000 deaths. (*3*) GAS affects communities worldwide, and its impact is modulated by the virulence of circulating strains and socioeconomic factors, such as household crowding. (*4*)

Invasive GAS (iGAS) infections occur when the bacterium enters a sterile location, such as blood, cerebrospinal fluid or deep tissues. (*5*) The global incidence has been estimated at 600 000 iGAS cases per year, causing more than 160 000 deaths. (*6*) However, the true burden is difficult to quantify, as many regions lack the facilities, resources and infrastructure to diagnose and monitor iGAS cases. (*7*)

Invasive infections are associated with high mortality, up to 20% within 7 days. (*8*) Groups at increased risk of iGAS infection include children younger than 2 years, pregnant women, adults aged 65 years and older, and individuals with comorbidities, such as diabetes or immunosuppression. (*2*) The incubation period of iGAS is difficult to define, but secondary iGAS cases have been reported up to 30 days after an initial case. (*5*)

Many countries have reported increases in iGAS incidence following the COVID-19 pandemic, potentially related to reduced exposure to iGAS during the pandemic, higher rates of circulating respiratory viruses and more virulent strains of iGAS. (*1*, *9*-*11*) Thus, management of iGAS is particularly relevant in this post-pandemic era.

IGAS has been notifiable in Queensland, Australia since 2005 and was made nationally notifiable in 2021. (*1*, *5*) The Series of National Guidelines (SoNG) for iGAS (version 1.0), from the Communicable Diseases Network Australia, was released in November 2023. (*5*) The purpose of this report is to outline the management of an iGAS outbreak in a rural residential aged-care facility in Queensland. This case study was used to examine the differences between the newly released SoNG and the previous Queensland Communicable Disease Control (QCDC) guidelines, and to explore the implications of the new guidelines on management in a rural context.

## Methods

### Setting

This outbreak occurred in rural Queensland. The residential aged-care facility includes six wings, housing more than 70 residents and employing 75 staff. Within the facility, four wings share communal areas and entrances (Area A). Two wings are geographically separate, with separate communal areas and entrances (Area B).

The local hospital is staffed by a generalist workforce who work across multiple specialties. Blood cultures are not processed locally but are transported to a regional laboratory, with molecular typing available at a metropolitan site that is more than 400 km from the facility.

### Outbreak description and epidemiological investigation

On 14 June 2023, the Darling Downs Public Health Unit received two iGAS notifications from a single residential facility. This met the QCDC criteria for an iGAS outbreak (i.e. two cases within 1 month). (*12*)

Both cases occurred in female residents in their 90s, and both subsequently died. Both cases were diagnosed in the local hospital. No other iGAS cases were identified within the region in the weeks preceding the outbreak.

Basic demographic data were entered into Queensland’s Notifiable Conditions System (NOCS). Enhanced surveillance data, including information about clinical presentation, risk factors and clinical outcomes, were collected through interviews with next of kin, facility staff or both. The investigation sought to identify the source of the outbreak via epidemiological evidence and liaising with local health professionals.

### Laboratory methods

#### Case definitions and confirmatory testing

A confirmed outbreak case was defined as any resident or staff member linked to the facility with culture or nucleic acid testing demonstrating GAS in a sterile site. (*5*) A contact was any resident or staff member who had resided or worked in the affected area during the 30 days before the outbreak. Visitors were not considered household-like contacts.

#### Genome sequencing

Whole genome sequencing was performed on all confirmed iGAS cases by the Public Health Microbiology Laboratory, Queensland Health. Sequences were trimmed using Trimmomatic v. 0.36. (*13*) Trimmed sequences were analysed using Kraken v. 1 for species identification. (*14*) Sequences were de novo assembled into contigs using SPAdes (St. Petersburg genome assembler) v. 3.12.0. (*15*) Multilocus sequence typing (MLST) and core genome MLST (cgMLST) analyses were performed in Ridom Seqsphere v. 9.0.8 using the pubMLST (public MLST) schemes. (*16*, *17*) A neighbour joining tree was generated from the cgMLST analysis using Ridom Seqsphere v. 9.0.8.

### Public health response

This outbreak occurred before the implementation of the SoNG for iGAS and thus was managed in line with the QCDC guidelines. An outbreak management team was convened, performed a risk assessment and identified potential contacts among residents and staff. The team liaised with local hospital infection control, facility and hospital staff, and local general practitioners (GPs). Information regarding cleaning, infection control and wound care was circulated to staff and residents. Staff, residents and families received written information about the risk of iGAS infection and outlining management recommendations.

Local hospitals were advised to increase testing and have a low threshold for treating suspected GAS or iGAS infection (e.g. any case of sepsis). The public health response was monitored by engaging weekly with the facility, liaising with emergency department clinicians, and reviewing cases potentially presenting with iGAS. Passive surveillance was undertaken through NOCS.

### Statistical analysis

Line lists were provided by the facility to monitor cases and the completion of chemoprophylaxis. Data were entered into a Microsoft Excel spreadsheet (v. 2305) to support a descriptive analysis of the outbreak.

## Results

### Epidemiological investigation

**Fig. 1:**
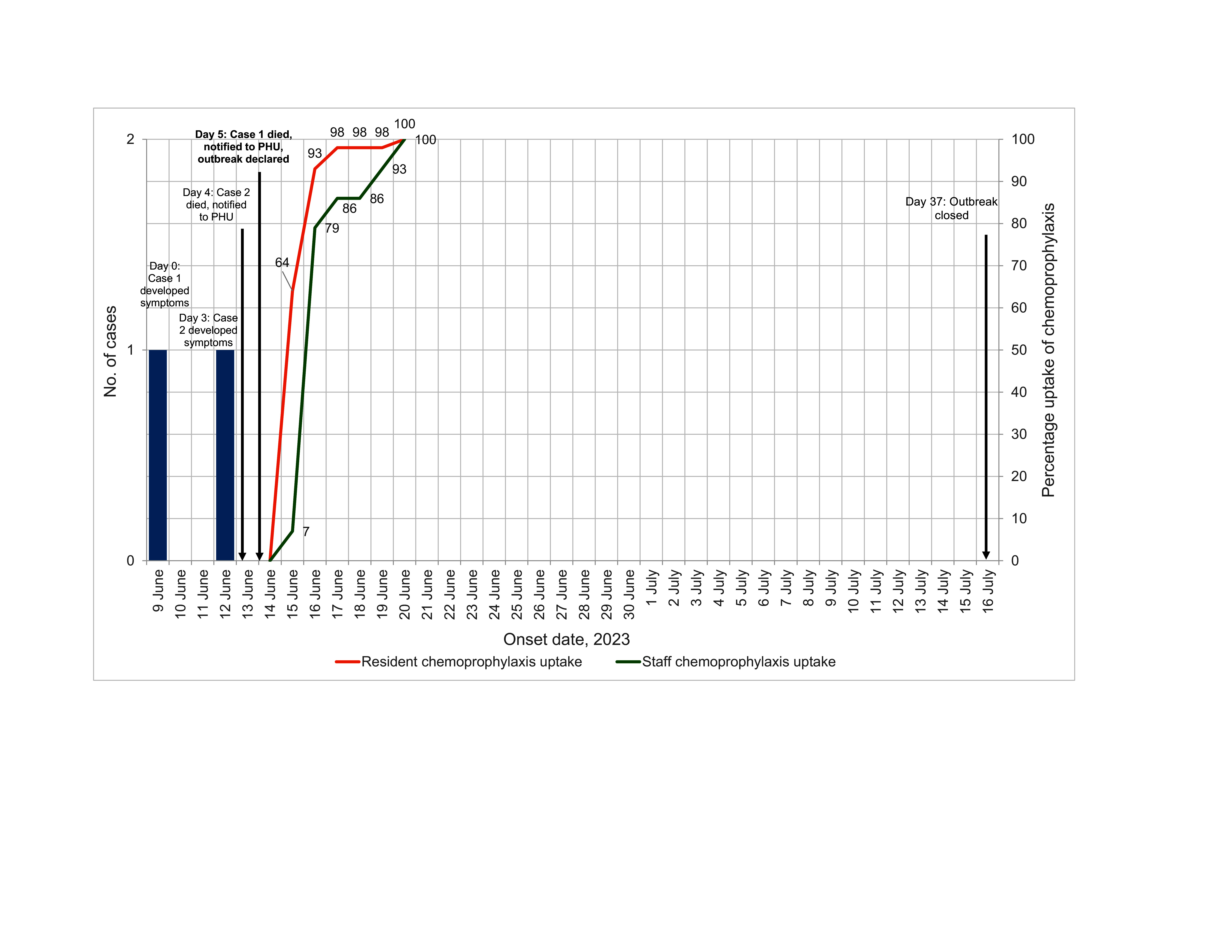
ETimeline of an outbreak of invasive group A Streptococcus (N = 2) and subsequent uptake of chemoprophylaxis by consenting residents (42/44) and staff (14/75) in a residential aged-care facility, Queensland, Australia, 2023

Both cases initially presented with cellulitis that progressed to bacteraemia and sepsis. The outbreak timeline is outlined in **Fig. 1**. Both residents lived in Area A, and thus that area was considered affected. No cases were identified in Area B.

[**insert** Figure 1]

Normal skin commensal bacteria were suspected to be the source of the outbreak, given the skin origin of both cases and the lack of other cases among staff or in the community.

### Laboratory investigation

Blood cultures from both cases were positive for GAS (genotype *emm4*). A phylogenetic tree based on whole genome sequencing with cgMLST analysis showed the two outbreak samples clustered closely together, with only a single allele difference.

### Public health response

#### Chemoprophylaxis

In line with the QCDC guidelines, chemoprophylaxis was recommended to all residents and staff who had resided or worked in Area A in the 30 days preceding the outbreak. (*12*) The risk assessment indicated that chemoprophylaxis was not required for hospital staff or patients.

Forty-four residents were identified as contacts, of whom 42 consented to chemoprophylaxis (95%). Treatment was provided without cost to residents by their usual GPs. One resident received benzathine benzylpenicillin, with the remainder receiving phenoxymethylpenicillin (*n* = 37) or cefalexin (*n* = 4).

Staff contacts were advised to seek chemoprophylaxis through private GPs, with the costs paid by staff. Consequently, the antibiotic they received was unknown. Fourteen of 75 total staff in the facility (19%) received chemoprophylaxis. The number of staff who worked in Area A during the exposure period is unclear, and thus the true coverage rate is unknown.

All residents and staff commenced chemoprophylaxis within 5 days of outbreak identification.

#### Surveillance and precautions

In line with the QCDC guidelines, facility and hospital surveillance for GAS and iGAS was increased, and a lower threshold for testing for and treatment of potential cases was adopted. Advice regarding facility cleaning, hand hygiene and wound care practices was provided to the facility and local hospital. No environmental health investigation was possible given the distance to the facility.

Surveillance continued for 30 days, and no additional cases were identified. The outbreak was declared over on 16 July 2023.

### Comparison with the new national management guidelines

Management of this outbreak, guided by the QCDC guidelines, largely aligned with the later-released SoNG for iGAS. The differences between the guidelines are summarized in **Table 1**.

**Table 1:** Comparison of the Queensland Communicable Disease Control guidelines with the Australian Series of National Guidelines (SoNG) for invasive group A *Streptococcus*

Category	Queensland Communicable Disease Control guidelines (*12*)	SoNG for invasive group A *Streptococcus* (*5*)	Would this have changed outbreak management?
**Terminology**	**Outbreak**	**Cluster**	**No**
**No. of linked cases required to define an outbreak or cluster**	**Two cases in 1 month**	**Two cases in 3 months**	**No; two cases were reported within 24 hours.**
**Additional information needed to confirm outbreak**	**Not applicable**	**Cases identical on molecular typing (unless cases are household contacts)**	**No; cases in the residential aged-care facility were classified as household contacts.**
**Duration of surveillance recommended post-outbreak**	**30 days**	**3 months**	**Yes**

#### Identification of outbreak or cluster

The change in guidelines did not impact the identification of the outbreak, since the cases developed symptoms within 3 days. The requirement for molecular typing when cases are not household contacts is new, but since the facility is considered a household-like setting, this change did not impact management. Thus, this facility met the outbreak criteria under both the QCDC guidelines and the SoNG.

#### Chemoprophylaxis

The QCDC guidelines recommended chemoprophylaxis in this case. (*12*) The SoNG similarly recommends chemoprophylaxis. (*5*)

#### Timeline for surveillance

The SoNG recommends post-outbreak surveillance for a minimum of 3 months, compared with 1 month in the QCDC guidelines. (*5*) Surveillance was ceased at 1 month for this outbreak, in line with the QCDC, although passive surveillance via NOCS continued. (*12*) Thus, a prolonged surveillance period would have been recommended under the SoNG.

## Discussion

This iGAS outbreak raised important issues related to the use of the SoNG in rural outbreaks. Many of these issues also relate to international settings with limited capacity for testing and public health action.

### Rural setting

This outbreak highlights the unique features of managing rural outbreaks. An advantage of the rural context is that the relatively small and interconnected medical services in these settings can streamline outbreak management.

However, rural workforce challenges may complicate management. Rural hospitals are commonly staffed by generalist clinicians who work across multiple settings. In this case, local hospital staff provided both general medical and obstetric care. Given the increased risk of secondary iGAS infection in birthing women and neonates, this was a significant consideration in the risk assessment. (*5*) While no significant risk was identified in this case, the unique context of rural hospitals must be considered.

Similarly, rural residential aged-care facilities experience greater workforce shortages than urban facilities, impacting their ability to cohort staff (i.e. assign staff to specific areas to minimize transmission risk) and recruit casual workers. (*18*) Similar workforce challenges are likely in many international settings.

Additionally, the lack of on-site pathology services may delay diagnosis. The requirement in the SoNG to confirm iGAS clusters by molecular typing is challenging in rural and international settings where typing is not easily accessible. Thus, in regions without rapid access to pathology services, public health staff may need to consider commencing management of a suspected outbreak before or without typing.

While standardized guidelines present the ideal management actions, it is vital to involve local professionals and consider the context during outbreak management.

### Duration of surveillance

The duration of post-outbreak surveillance changed under the SoNG for iGAS. The QCDC guidelines recommended 1 month of surveillance. In comparison, the SoNG for iGAS recommends at least 3 months of post-outbreak surveillance, bringing Australia more in line with American guidelines. (*19*) In this outbreak, passive surveillance via NOCS continued, and it did not identify any additional iGAS cases. However, lengthening the post-outbreak surveillance period would potentially allow for increased staffing and laboratory resources, and other support over a longer period.

### The role of chemoprophylaxis

Both the QCDC guidelines and the SoNG supported chemoprophylaxis in this case. This agreement notwithstanding, the role of chemoprophylaxis in institutional iGAS outbreaks remains unclear. A 2012 review of iGAS outbreaks in care homes in England found that widespread chemoprophylaxis had no benefit in controlling the spread of the outbreak. (*20*) However, these findings were complicated by a lack of consistency in chemoprophylaxis regimens and thresholds. (*20*)

Regardless of whether chemoprophylaxis is used, infection control and wound care practices have important roles in managing iGAS outbreaks, and these practices are often significant causative factors in outbreaks. (*8*, *21*) In the outbreak reported here, a skin infection was the source of infections in both residents, underscoring the importance of infection control within a vulnerable elderly population.

In this case, chemoprophylaxis was considered a useful adjunct to other outbreak control measures (e.g. infection control) to protect a vulnerable population. In urban settings, alternative approaches, such as staff cohorting and swabbing, may be more feasible. Considering the local context is therefore vital to decision-making.

### Limitations

This case study describes a single, small outbreak in a residential aged-care facility. It was managed in accordance with the QCDC guidelines. The SoNG was not released until after the outbreak had ended, and thus post-outbreak surveillance was not in line with the SoNG recommendations, except for the passive surveillance through NOCS. In addition, the distance to the facility prohibited additional assessments (e.g. environmental health), which may have been valuable.

### Conclusions

This report outlines the management of an iGAS outbreak within a rural residential aged-care facility. It highlights the importance of considering contextual factors in outbreak management. These findings are also significant for lower-resource settings, where limitations in the workforce, laboratory services and finances may make standard outbreak management difficult to achieve.
